# Diet diversity and nutritional status among adults in southwest China

**DOI:** 10.1371/journal.pone.0172406

**Published:** 2017-02-23

**Authors:** Qiang Zhang, Xinguang Chen, Zhitao Liu, Deepthi S. Varma, Rong Wan, Shiwen Zhao

**Affiliations:** 1 Department of Nutrition and Food Hygiene, Yunnan Center for Disease Control and Prevention, Kunming, China; 2 Department of Epidemiology, University of Florida, Gainesville, Florida, United States of America; State University of Rio de janeiro, BRAZIL

## Abstract

**Background:**

With rapid urbanization in the past decades, diet diversity continues to increase in China. The present cross-sectional study aims to explore the association between dietary diversity and nutritional status among adults in southwest China.

**Methods:**

This study used data from 2011–2012 National Nutritional Survey in Yunnan Province, southwest China (N = 1105).Data of three consecutive 24-hour dietary recalls were used to calculate dietary diversity scores (DDS) and nutrient adequacy ratio (NAR). Body mass index and waist circumference were used to determine nutritional status. Surveylogistic procedure of SAS 9.2 software was used to examine the association between DDS and obesity by estimating odds ratio (OR) and 95% confidence intervals (CI).

**Results:**

The mean DDS was 5.2 (SD 1.1) out of nine points. Being female, younger age, belonging to Han ethnicity, having higher educational level and household income were positively associated with DDS (all P<0.05). As DDS increased, consumption also increased in most food groups except grains and vegetables. People with medium and high DDS (DDS = 5 and DDS ≥6, respectively) ingested more energy than the recommended quantity(NAR = 1.1 and 1.2, respectively). However, the intakes of Calcium and Vitamin A were seriously inadequate even for people with high DDS (NAR≤0.5). With potential confounders adjusted, people with medium and high DDS were at higher risk of general and central obesity than people with DDS ≤4 (OR = 1.4–1.9, P<0.01).

**Conclusions:**

Our data indicated that high DDS was associated with excessive energy intake and obesity among adults in southwest China. Although dietary diversity is widely recommended, public health messages should give less emphasis on dietary diversity.

## Introduction

Obesity has become a major global health challenge, contributing to the rapid growth of chronic diseases such as diabetes, cancer and cardiovascular diseases [1,2]. Between 1991 and 2011, among Chinese adults, the prevalence of general obesity dramatically increased from 3.5% to 11.8%, and has doubled from 20.4% to 44.0% for central obesity [3,4]. This trend is driven by a range of factors, especially a remarkable shift from traditional diets to high-fat and high-energy diets over the past decades [5].

Traditional dietary analyses have had certain limitations because they have focused merely on the relationship between individual nutrients or foods and obesity [6,7]. In recent years, dietary indices have emerged as an alternative, holistic approach [8]. The dietary diversity score (DDS) is one of the a priori defined dietary indices used to assess overall diet quality [9]. Dietary diversity has been known as a key index of high diet quality in various populations [10–12]. Nevertheless, the association between dietary diversity and obesity remains controversial across different cultures. Although higher DDS is associated with increased intake of fiber and vitamin C [13], which are protective factors against obesity, eating a more varied diet is usually associated with higher energy intake [14,15]. Studies in Sri Lanka and Brazil suggested that dietary diversity was positively associated with obesity [16,17]. However, studies in the US and Iran showed an inverse association between dietary diversity and obesity [18,19]. Furthermore, no such significant associations were found in other studies [20,21]. In addition to the differences in dietary intake assessment and DDS determination, culturally specific dietary habits are likely to have a role [22]. These findings have limited their applicability to Chinese population.

As a less developed geographical area, the prevalence of obesity has traditionally been lower in southwest China [23,24]. But with economic development and urbanization in recent years, dietary habits have changed rapidly which raises great concern regarding obesity and other health outcomes [25]. In this study, we examined the association between DDS and obesity using the latest data from National Nutrition Survey in southwest China. Moreover, food consumptions and nutrient adequacy were also analyzed to further understand the correlation of DDS and obesity.

## Materials and methods

### Ethics statement

This study was approved by Institutional Review Board at the China Center for Disease Control and Prevention. Signed informed consent was obtained from all the participants before the survey.

### Study population

Data for the present study were from the 2011–2012 National Nutritional Survey in Yunnan Province, southwest China. Participants were recruited using a stratified cluster sampling method from four counties which were randomly selected from Yunnan Province. From each of them, three townships were randomly selected. Then two villages were further randomly selected from each township. In each village, thirty households were randomly selected. All members in the households were invited to take part in the survey. Altogether, 1202 participants aged 18 years and over completed the survey, with a response rate of 96.0%. Ninety seven participants were excluded because of pregnancy (24 subjects), being on a prescribed diet (25 subjects), serious illness (16 subjects) or extreme energy intake (<1000kcal or >4000kcal, 32 subjects). The final ana1ysis has been conducted on 1105 participants.

### Dietary survey

Trained interviewers from local Centers for Disease Control and Prevention visited the selected households to collect the information on food consumptions using three consecutive 24-h dietary recalls (including two weekdays and one weekend day). Socio-demographic characteristics, lifestyle and physical activity of participants were also recorded using pre-coded questionnaires. Energy and nutrient intakes were calculated using the data of dietary records in conjunction with the China Food Composition Table [26]. Nutrient Adequacy Ratio (NAR) was used to determine the adequacy of energy, protein and other 14 micronutrients in diet. NAR for a given nutrient was the ratio of a participant’s intake to the Recommended Nutrient Intakes (RNI) in Chinese Dietary Guidelines.

### Dietary diversity score

DDS was defined as the number of food groups consumed over the 3 days based on the dietary recalls. According to the Chinese Dietary Guidelines, all food items were categorized into 9 groups which were grains (including cereals, tubers, and roots), vegetables, fruits, meat (including pork, beef, poultry and organs), beans (including beans, nuts and seeds), eggs, fish (including seafood, freshwater fish and aquatic products), dairy (including milk and products), and oil (including animal and vegetable oil). If a participant consumed any food from any of the above mentioned categories, he would get one point in that food category. Otherwise, he would be scored zero. Consuming different foods from the same category would not count repeatedly. Total score was the sum scores of the nine food groups and the maximum score could go up to 9.

### Anthropometric measures

Well trained local health workers collected anthropometrical measurements following a reference protocol recommended by the World Health Organization [27]. Body weight was measured by using platform scales to the nearest 0.1 kilogram (kg), without heavy clothes. Height was measured by using a height chart to the nearest 0.1 centimeter (cm), without shoes. Body Mass Index (BMI) was calculated as weight (kg) divided by the square of height (m^2^). Waist circumference (WC) was measured to the nearest 0.1 cm at the level of the iliac crest while the participant was at normal respiration. For each participant, WC was measured twice and the average was recorded. This study adopted the Chinese standard for Obesity as proposed by the Working Group on Obesity in China (WGOC). General obesity was defined as BMI ≥28.0 and central obesity was defined as WC ≥85.0 cm or WC ≥80.0 cm for men and women respectively [28].

### Definition of other variables

“Low education” was defined as middle school and below, while “high education” was defined as high school and above. Income was measured by household income per capita in the last 12 months. Smoking status was defined as current smokers and non-current smokers. Current smokers were people smoking any tobacco products at the time of the survey while non-current smokers were not [29]. Alcohol intake was defined as non-drinker, moderate drinker (≤2 times/week) and heavy drinker (>2 times/week) [30]. The level of work, leisure, commute and household work of participants in the last 12 months were assessed using a physical activity questionnaire. Physical activity level was measured by Metabolic Equivalent Task (MET) hours per week [31]. Ethnic groups were categorized as Han and minorities because a large majority of population that lives in southwest China are ethnic minorities with low obesity prevalence as compared to Han ethnic [32,33].

### Statistical analysis

Considering the fact that the data were extracted by the stratified cluster sampling method, analyses were done using a survey procedure. Surveyfreq was used to calculate socio-demographic characteristics of the study population. Surveymeans was used to compare differences of dietary diversity score according to socio-demographic characteristics. Numeric variables (e.g. DDS and NAR) were presented as Mean ± SD, while categorical variables (e.g. gender) were presented as percentage (%). Food consumptions which did not follow normal distribution were presented as median and quartile range. Logistic regression models with potential confounders adjusted were developed to test the correlation of dietary diversity with general and central obesity by estimating odds ratio (OR) and 95% confidence intervals (CI) using Surveylogistic procedure. All statistical analyses were performed with SAS 9.2 (SAS Institute, Cary, NC).

## Results

Table 1 showed the socio-demographic characteristics and DDS of the 1105 participants. The total sample comprised 40% males and 60% females (weighted: 50.4% and 49.6%, respectively). Participants aged 18–59 years, 60 years and above accounted for 81.5% and 18.5%, respectively. More than half (53%) of the participants were minorities. Female gender, younger age, Han ethnicity, higher income and education levels were positively associated with DDS. No significant difference was found between DDS and marital status.

**Table 1:** Socio-demographic characteristics and dietary diversity score of the participants.

	N	Unweighted (%)	Weighted (%)	DDS		
				Mean	*SD*	*P value*
AllGender	1105	100	100	5.2	1.1	
Sex						
Men	444	40.0	50.4	5.1	1.0	<0.01
Women	661	60.0	49.6	5.3	1.1	
Age (years)						
18~	906	81.5	85.3	5.2	1.1	<0.01
60~	199	18.5	14.7	4.8	1.0	
Marital status						
Married	1002	90.7	89.3	5.2	1.1	0.1
Unmarried	103	9.3	10.7	5.0	1.0	
Ethnic group						
Han	519	47.0	46.3	5.3	1.0	<0.01
Minorities	586	53.0	53.7	5.1	1.1	
Education level						
Low	790	71.7	68.9	5.1	1.0	<0.01
High	315	28.3	31.1	5.4	1.0	
Income (Yuan)						
<5000	767	69.6	68.7	5.1	1.0	<0.01
10000-	338	30.4	31.3	5.3	1.1	

The DDS of the 1105 participants were found to be normally distributed in this study (Mean = 5.2, SD = 1.1), ranged between 2 and 8. No one consumed foods in the nine groups and no one consumed foods less than two groups. Participants were divided into 3 groups by DDS for further analysis: Low, Medium, and High. The low DDS group included participants with DDS ranging from 2 to 4, the medium DDS group included participants with DDS equaling 5, and the high DDS group included participants with DDS ranging from 6 to 8. There were 279 (25.2%), 421 (38.1%) and 405 (36.7%) participants in the low, medium and high DDS group, respectively.

Table 2 showed dietary intakes of the participants by DDS groups. Compared with the Chinese Dietary Guidelines, consumption of grains, meat and oil were excessive, while consumption of other six food groups were inadequate. As DDS increased, consumption increased in most food groups except grains and vegetables. In particular, people with medium and high DDS groups nearly consumed 50% more meat and oil than the recommendations. However, the increase of dairy, fish and fruits consumption were limited. Although the percentage of energy from fat increased with DDS, it did not exceed the recommendation in the three groups.

**Table 2:** Dietary intakes of the participants by dietary diversity score groups.

	DDS			*Recommendations*
	Low (2–4)	Medium (5)	High (6–8)	
	(N = 279, 25.2%)	(N = 421, 38.1%)	(N = 405, 36.7%)	
Food groups (g/d)				
Grains	523 (41, 681)	520(401, 705)	520(400, 678)	200–400
Vegetables	197(122, 297)	189(119, 295)	183 (104, 295)	300–500
Fruits	0 (0, 0)	0 (0, 0)	46(0, 140)	200–400
Beans	0 (0, 0)	0(0, 25)	15 (4, 34)	30–50
Meats	77(0, 173)	114 (65, 188)	116 (68, 176)	50–75
Dairy	0 (0, 0)	0 (0, 0)	0 (0, 0)	300
Eggs	0 (0, 0)	0 (0, 16)	18(0, 38)	25–50
Fish	0 (0, 0)	0 (0, 0)	0 (0, 19)	50–100
Oil	23(13, 45)	33(17, 52)	36 (19, 58)	25
Energy from fat (%)	18.5	22.5	24.5	<30

Note: The amounts of food intakes are expressed as Median (quartile range)

Table 3 shows the NARs of 16 individual nutrients in the participants’ diet by DDS groups. Most NARs increased with DDS, except Vitamin C and sodium. NARs of energy, protein and other six nutrients (Phosphor, Sodium, Iron, Niacin, Zinc and Vitamin E)in all groups were closer to or higher than 1.0, while NARs of other six nutrients (Magnesium, Potassium, Thiamine, Riboflavin, Vitamin C and Selenium) ranged from 0.5 to 1.0. The NARs of Calcium and Vitamin A were the lowest, which were only 0.5 even for the high DDS group.

**Table 3:** NARs of 16 individual nutrients in the participants’ diet by dietary diversity score groups.

	DDS			*P value*
	Low (2–4)	Medium (5)	High (6–8)	
NAR				
Energy	1.02±0.32	1.13±0.34	1.18±0.33	<0.01
Protein	0.94±0.33	1.12±00.36	1.23±0.39	<0.01
P	1.21±0.40	1.37±0.40	1.51±0.43	<0.01
Sodium	2.33±1.25	2.35±1.27	2.49±1.37	0.21
Fe	1.09±0.47	1.35±0.50	1.47±0.52	<0.01
Niacin	1.09±0.43	1.28±0.50	1.32±0.54	<0.01
Zn	0.95±0.33	1.06±0.33	1.13±0.36	<0.01
Vitamin E	0.99±0.88	1.43±1.22	1.86±1.43	<0.01
Mg	0.80±0.29	0.88±0.27	0.97±0.30	<0.01
K	0.72±0.30	0.83±0.32	0.96±0.34	<0.01
Thiamine	0.73±0.32	0.82±0.32	0.87±0.33	<0.01
Riboflavin	0.44±0.17	0.52±0.20	0.60±0.24	<0.01
Vitamin C	0.75±0.47	0.72±0.43	0.87±0.46	0.72
Se	0.48±0.21	0.50±0.32	0.78±0.32	<0.01
Vitamin A	0.32±0.29	0.50±0.32	0.50±0.33	<0.01
Ca	0.26±0.12	0.30±0.13	0.37±0.17	<0.01

Note: Values are expressed as Mean±SD.

Table 4 showed the OR and 95% CI for having general and central obesity across the three DDS groups. With potential confounders adjusted, DDS were positively associated with general and central obesity. People in the high DDS group had higher risk of general obesity (OR = 1.9; 95%CI 1.1–3.7) and of central obesity (OR = 1.9; 95% CI 1.3–2.8).

**Table 4:** Multivariate-adjusted odds ratio and 95% CI for having general obesity and central obesity.

	DDS			*P for trend*
	Low (2–4)	Medium (5)	High (6–8)	
General obesity				
Model 1	1.0	2.1(1.1–4.0)	2.2 (1.2–4.0)	<0.01
Model 2	1.0	2.0 (1.0–3.8)	2.1 (1.1–3.9)	<0.01
Model 3	1.0	1.7 (0.9–3.3)	1.9 (1.1–3.7)	<0.01
Central obesity				
Model 1	1.0	1.5(1.1–2.2)	2.2 (1.5–3.1)	<0.01
Model 2	1.0	1.(1.1–2.2)	2.1(1.4–3.1)	<0.01
Model 3	1.0	1.4(1.0–2.1)	1.9 (1.3–2.8)	<0.01

Note: Model 1: adjusted for age, sex, ethnic groups, education and income. Model 2: model 1 with additional adjusted for energy intake and physical activity. Model 3: model 2 with additional adjusted for smoke and drink.

## Discussion

With the latest cross-sectional data from National Nutrition Survey in southwest China, findings of this study indicated positive associations between DDS and general obesity as well as central obesity in adults. Findings of this study highlighted the increasing risk of obesity due to unbalanced diets along with increasing dietary diversity. This study also showed the inadequate intake of some important micronutrients even for participants with high DDS.

Dietary imbalance coming with dietary diversity is an important cause for increasing obesity. Similar to other developing countries, China experiences an ongoing nutrition transition [34]. Favorable and unfavorable nutrition changes have interacted in this process. A national representative study indicates that between 1989 and 2006, consumptions of most foods (except cereals) were consistently increased in Chinese adults and the most prominent ones were meat and oil [35]. In this study, the consumptions of meat and oil increased significantly with DDS, which were nearly 50% over recommendations for people with medium and high DDS. In contrast, the consumptions of dairy, fish and fruits were far below recommendations and increased little with DDS. Due to limited food supply and nutrition knowledge, unbalanced dietary practices are more prevalent in less developed areas [36]. Therefore, nutritional education and interventions on diet balance are necessary in southwest China.

Varied but unbalanced diets fail to provide rational nutrient supply. In the present study, NAR for most nutrients were positively correlated with DDS, which was consistent with other studies [9,13]. In particular, no significant association between the NAR of Vitamin C and DDS was detected in this study. This could be due to the different levels of vegetables and grains intakes in the three groups. Our study indicates that energy and protein deficiency is no longer a prominent nutritional problem in southwest China, instead, the inadequate intake of micronutrients deserves further attention. In this study, NAR of Ca and Vitamin A in people with high DDS were 50% below the recommendations. In general, diet in southwest China is shifting towards a high-fat, high energy-density and poor-micronutrients dietary pattern, which is similar to the findings of another study from China [37].

However, some studies reported inverse associations between DDS and obesity. A study among female college students in Tehran showed that DDS was related to increasing consumption of healthy foods, such as fruit, vegetables and whole grains instead of meat [19]. Another study in Belgium found that DDS was positively associated with dietary balance [11], which is different from what we have found in this study. These findings suggest that the transition of dietary diversity may be different in different cultures, and therefore the health impact of dietary diversity should be analyzed specific to each culture.

The present study has several limitations. First, this study used a cross-sectional data. Therefore, causal relations cannot be determined. Second, the 24-h dietary recall method cannot generally evaluated usual dietary intake. Third, health values of consumed of food items used to measure healthy food diversity have not considered. Fourth, physical activity data among the participants was not considered to calculate daily energy requirements. Finally, the data for this analysis were only collected from four counties in southwest China. Therefore, caution is needed to generalize these findings to other places in China, especially in the East.

## Conclusions

The findings of this study revealed that dietary diversity was positively associated with general and central obesity among adults in southwest China, and that excessive energy intake and micronutrient deficiencies of varied diets. Although dietary diversity is widely recommended, public health messages should emphasize to improve moderate and balanced dietary diversity in selected food items.
